# Optimization of Ultrasonic-Assisted Extraction of Total Phenolics from *Citrus aurantium* L. Blossoms and Evaluation of Free Radical Scavenging, Anti-HMG-CoA Reductase Activities

**DOI:** 10.3390/molecules24132368

**Published:** 2019-06-26

**Authors:** Kexin Hao, Wenzhong Hu, Mengyang Hou, Duo Cao, Yu Wang, Qingxin Guan, Xiufu Zhang, Aosheng Wang, Jiaoxue Yu, Binmei Guo

**Affiliations:** 1 College of Life Science, Dalian Nationalities University, Dalian 116600, China; 2 Key Laboratory of Biotechnology and Bioresources Utilization, Ministry of Education, Dalian 116600, China; 3 School of Life Science and Biotechnology, Dalian University of Technology, Dalian 116024, China; 4 College of Life Science, Northwest University, Xi’an 710069, China

**Keywords:** *Citrus aurantium* L. blossoms, total phenolics, ultrasonic-assisted extraction, Box–Behnken design, free radical scavenging activity, anti-HMG-CoA reductase activity

## Abstract

The objective of this study was to develop an ultrasonic-assisted procedure for the extraction of total phenolics from *Citrus aurantium* L. blossoms (CAB) and evaluate the free radical scavenging activity and anti-HMG-CoA reductase activity of the total phenolics. In this work, a Box– Behnken design based on single-factor experiments was used to explore the optimum extraction process. Under the optimum conditions (extraction solvent 70.31% ethanol, extraction temperature 61.94 °C, extraction time 51.73 min, and liquid-to-solid ratio 35.63 mL/g), the extraction yield of total phenolics was 95.84 mg gallic acid equivalents (GAE)/g dry matter (DM), which was highly consistent with the theoretical value (96.12 mg GAE/g DM). The higher contents of total phenolics and five main phenolic compounds obtained from the optimized ultrasonic-assisted extraction (UAE) proved its efficiency when compared with conventional heat reflux extraction (HRE). The total phenolic extract showed excellent free radical scavenging properties against 1,1-diphenyl-2-picrylhydrazyl radical (DPPH·), 2,2′-azino-bis-(3-ethylbenzthiazoline-6-sulphonic acid) radical (ABTS^+^·), hydroxyl radical (·OH) and superoxide anion radical (·O_2_^−^), with IC_50_ values of 197.007, 83.878, 218.643, and 158.885 μg/mL, respectively; the extracts also showed good inhibition of β-hydroxy-β-methylglutaryl-CoA reductase (HMG-CoA reductase) activity, with an IC_50_ value of 117.165 μg/mL. Total phenolics from CAB could be a potential source of natural free radical scavenger and HMG-CoA reductase inhibitor.

## 1. Introduction

Free radicals, atoms or groups containing unpaired electrons, are fairly active, which is essential for any physiological metabolism of an organism [1]. As is well known, an organism itself has the ability to balance free radicals, but the risk of several serious diseases will increase if the organism cannot get rid of excess free radicals. Modern medical studies have demonstrated that free radicals can lead to DNA, protein and lipid oxidative damage and have a direct relationship with cancer, cardiovascular diseases, Alzheimer’s and Parkinson’s disorders [2,3,4,5,6,7]. Dietary supplementation of exogenous free radical scavengers, is associated with a reduced incidence of those frightening diseases. Despite the enormous development of chemical synthesis, many excellent natural free radical scavengers with fewer side effects from edible or medicinal plants have been successfully exploited over the past several years [8]. Plants are rich in various bioactive compounds, in which phenolics have attracted increasing attention due to their pre-eminent free radical scavenging property.

In recent years, the morbidity and mortality of cardiovascular and cerebrovascular diseases have risen dramatically due to the improvement of people’s living standards [9]. These diseases have very high correlation with hyperlipidemia, mainly manifesting as high levels of cholesterol in the blood [10]. A key regulatory enzyme, 3-hydroxy-3-methylglutaryl-coenzyme A reductase (HMG-CoA reductase), has a significant impact on cholesterol biosynthesis [11]. Hence, an effective method for reducing cholesterol level is to inhibit HMG-CoA reductase activity. The existing HMG-CoA reductase inhibitors are mainly statin drugs. Unfortunately, other potentially harmful side effects, such as myopathy and liver damage, appear with long-time use of statin drugs [12,13]. Therefore, the screening of novel HMG-CoA reductase inhibitors is urgent.

*Citrus aurantium* L. belonging to the genus *Citrus* (Rutaceae), is mainly cultivated in tropical and subtropical areas. *Citrus aurantium* L. is a well-known medicinal and edible plant in China and has been traditionally used for treatment of obesity, indigestion, chest congestion, bellyache, nausea, and vomiting [14], as well as recently attracting attention for its extensive biological activities including anticancer, antibacterial, antioxidant, antidiabetic, enhancing immunity, and treatment for neurological disorders [15,16,17,18,19]. *Citrus aurantium* L. blossom (CAB) especially has been popularly consumed in a variety of functional beverages for a long time in China. Recently, CAB has attracted increasing attention because of its extensive biological activities including anti-complement, anti- inflammatory, anti-tumor, and antioxidant activities [15,16,17]. In addition, previous studies have shown that CAB is a good source of phenolic compounds. For example, Shen et al. found that the crude polyphenols extracted from CAB mainly contained neoeriocitrin, eriocitrin, rhoifolin, hesperidin, naringin, rutin, neohesperidin, and hesperetin by LC-MS analysis [15]. Karimi et al. also found that CAB contained pyrogallol, caffeic acid, gallic acid, and syringic acid by RP-HPLC analysis [16]. The phenolic compounds were the major contributors to the beneficial health effects of CAB.

In fact, extraction technology not only has a direct influence on the extraction efficiency of phytochemical components, but also has a profound effect on the biological activities. Karimi et al. researched the effects of solvent type on phenolics and flavonoids contents and anti-tumor, anti-inflammatory, antioxidant activities of CAB [16]. Yang et al. optimized the extraction process of flavonoids from CAB [18]. Although the optimum conditions for the extraction of polyphenols from CAB with water as the extraction solvent were studied by Shen et al. [19], the optimized extraction process required a high temperature (90 °C) and took a long time (each time for 1 h), moreover, according to the result reported by Karimi et al. [16], not only did the aqueous extract exhibit the lowest DPPH· scavenging activity, but the extraction yield of water was the lowest compared with methanolic, and ethanolic extracts. Hence, there is an urgent need to develop a novel and efficient approach to extract phenolic substances from CAB. Compared with conventional solvent extraction methods (cold or warm soaking, decoction, heat reflux, and Soxhlet extraction), ultrasonic-assisted extraction (UAE) is relatively simple, efficient, and practical. UAE can also enhance the biological activities of extracts [20,21,22]. As a novel technology, UAE has been proposed by many researchers, such as for phenolic compounds from blueberry pomace [23], *Oryza sativa* L. ‘Violet Nori’ [24], *Brosimum alicastrum* leaves [25], and *Acer Truncatum* leaves [26].

This study was committed to develop an energy-efficient technology for extraction of total phenolics from CAB by UAE, and the process parameters were optimized through single-factor experiment and Box–Behnken design. In addition, free radical scavenging and anti-HMG-CoA reductase activities of total phenolic extract were evaluated in vitro.

## 2. Results and Discussion

### 2.1. Single-Factor Experimental Analysis

#### 2.1.1. Effect of Ethanol Concentration on the Extraction Yield of Total Phenolics

**Figure 1:**
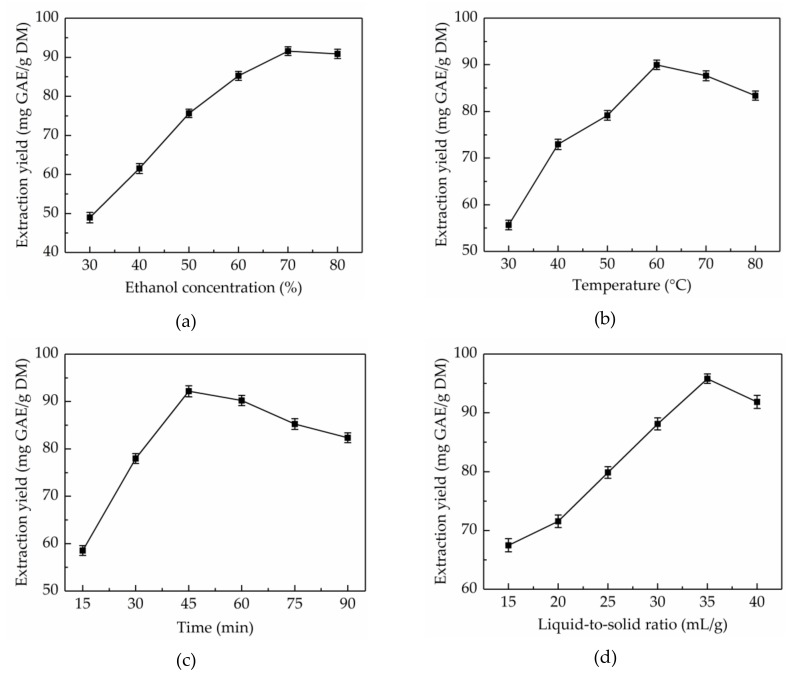
Effects of ethanol concentration (**a**), extraction temperature (**b**), extraction time (**c**), and liquid-to-solid ratio (**d**) on the extraction yield of total phenolics.

Aqueous ethanol solution is a most common extraction solvent, which has been extensively used for the extraction of natural products due to its high-efficiency, and eco-friendly and recyclable characteristics. The extraction solvent with proper polarity is vital to the solubility of total phenolics [27]. Different concentrations of ethanol have different polarity, therefore, the effects of ethanol concentrations (30–80%) on the extraction yield of total phenolics from CAB were evaluated when controlling for other factors (extraction temperature 45 °C, extraction time 30 min and liquid-to-solid ratio 30 mL/g). As shown in Figure 1a, the extraction yield of total phenolics significantly improved from 48.95 ± 1.32 to 91.58 ± 1.12 mg GAE/g DM with the increase in ethanol concentration from 30% to 70%. However, the extraction yield declined with further increase of ethanol concentration. Hence, 70% ethanol was considered as the optimal extraction solvent.

#### 2.1.2. Effect of Extraction Temperature on Extraction Yield of Total Phenolics

In general, the higher the extraction temperature, the higher is the extraction yield, which is due to the fact that molecules move faster at higher temperature, leading to enhanced diffusion and permeation behaviors [28]. However, if the extraction temperature is too high, the heat-sensitive components would be destroyed. Hence, to improve extraction yield, extraction temperatures in the range of 30–80 °C were studied when controlling for other factors (ethanol concentration 70%, extraction time 30 min, and liquid-to-solid ratio 30 mL/g). Figure 1b showed that the extraction yield of total phenolics from CAB obviously increased in the range of 30–60 °C, however, further increase in the extraction temperature resulted in a gradual decrease in the extraction yield. The highest extraction yield of total phenolics (89.98 ± 1.01 mg GAE/g DM) was obtained at 60 °C. Therefore, 60 °C was considered as the optimum extraction temperature.

#### 2.1.3. Effect of Extraction Time on Extraction Yield of Total Phenolics

Time is a significant factor influencing the extraction yield of total phenolics. A reasonable extraction time can help shorten the production cycle, reduce energy consumption, and improve yield. However, too long an extraction time will result in the decomposition of the target compounds [29]. To optimize the extraction time, the extraction time was set at 10–60 min, meanwhile, other variables were kept constant (ethanol concentration 70%, extraction temperature 60 °C, and liquid-to-solid ratio 30 mL/g). Figure 1c shows that the extraction yield of total phenolics from CAB increased with time and reached the highest (92.16 ± 1.15 mg GAE/g DM) when the extraction time was 45 min, and after this point in time, the extraction yield started to decline. Thus, the ultrasonic time was maintained at 45 min for the next step.

#### 2.1.4. Effect of Liquid-to-Solid Ratio on Extraction Yield of Total Phenolics

The liquid-to-solid ratio has a significant role in the mass transfer of extracts between materials and extraction solvents [30]. To obtain the ideal extraction yield of total phenolics, selecting a suitable liquid-to-solid ratio is of the essence. The probable reason is that total phenolics from CAB cannot be fully extracted in the case of low liquid–solid ratio, conversely, when the liquid–solid ratio is too high, the content of impurity and operational cost increases. To optimize the liquid–solid ratio, in this work, the liquid–solid ratio was set at 15–40 mL/g, meanwhile, other variables were kept constant (ethanol concentration 70%, ultrasonic temperature 60 °C, and extraction time 45 min). As shown in Figure 1d, the extraction yield of total phenolics from CAB increased from 67.49 ± 1.12 to 95.81 ± 0.79 mg GAE/g DM when the liquid-to-solid ratio increased from 15 to 35 mL/g. However, on further increasing the liquid-to-solid ratio the extraction yield of total phenolics decreased. Thus, 35 mL/g was regarded as the optimal liquid-to-solid ratio.

### 2.2. Optimization of Variables by Box–Behnken Design

#### 2.2.1. Statistical Analysis and Model Fitting

Single-factor experiments can only study the effects of changes in one factor on the response variable. The truth, however, is that the influence of variables on response variables is interdependent and mutually restricted. Hence, the influence of the interaction between the main four variables (ethanol concentration, extraction temperature, extraction time, and liquid-to-solid ratio) on extraction yield of total phenolics was researched by the Box–Behnken design. Detailed experimental design and results are summarized in Table 1.

Multiple regression analysis was used to determine the correlation of the four variables and the extraction yield of total phenolics, and a second-order polynomial equation yielded was represented as below:

(1) $$\begin{array}{ll} Y= & -1309.09267+18.69873X_{1}+13.83737X_{2}+2.95973X_{3}+13.61713X_{4}-0.008975X_{1}X_{2}\\ & -0.00263333X_{1}X_{3}-0.0344X_{1}X_{4}+0.01005X_{2}X_{3}+0.0382X_{2}X_{4}\\ & -0.021267X_{3}X_{4}-0.11931X_{1}{}^{2}-0.12178X_{2}{}^{2}-0.025506X_{3}{}^{2}-0.17491X_{4}{}^{2} \end{array}$$

where X_1_, X_2_, X_3_ and X_4_ represent the ethanol concentration, extraction temperature, extraction time, and liquid-to-solid ratio, respectively. *Y* represents the extraction yield of total phenolics.

The parameters acquired from the analysis of variance (ANOVA) for the Box–Behnken design are listed in Table 2. A model with an *F*-value of 66.3436 and *p*-value of <0.0001 meant that the model was highly significant. It also meant this model was very consistent with experimental data. The *F*-value and *p*-value of the lack of fit were 2.6222 and 0.1829, respectively, which meant that the lack of fit was not significant relative to the pure error. The coefficient (*R*^2^ = 0.9852) meant this model could account for 98.52% of the response value changes and the fitting precision of this model was satisfactory. The adjusted coefficient (Adj. *R*^2^ = 0.9703) was close to the *R*^2^, which meant that the experimental values fitted well with the predicted values. The value of the coefficient of variation (C.V%) was very low (1.91), which meant the model was repeatable [31]. In addition, the linear coefficients (X_1_, X_2_, X_3_, and X_4_), cross product coefficients (X_1_X_4_ and X_2_X_4_), and quadratic coefficient (X_1_^2^, X_2_^2^, X_3_^2^, and X_4_^2^) were significant (*p* < 0.05).

#### 2.2.2. Optimization of UAE Procedure

**Figure 2:**
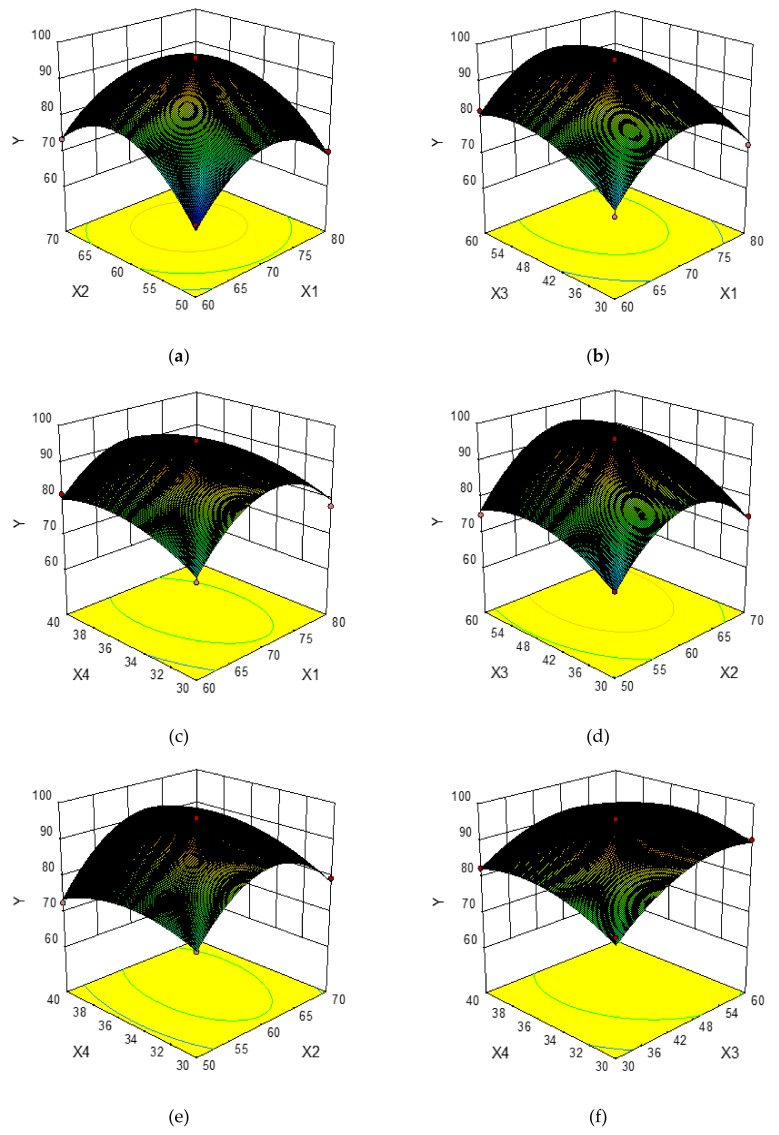
Response surface plots showing the effects of variables on the extraction yield of total phenolics: interaction of the ethanol concentration and extraction temperature (**a**), interaction of ethanol concentration and extraction time (**b**), interaction of ethanol concentration and liquid-to-solid ratio (**c**), interaction of extraction temperature and extraction time (**d**), interaction of extraction temperature and liquid-to-solid ratio (**e**), interaction of extraction time and liquid-to-solid ratio (**f**).

In order to provide a better visual representation of the effects of interactions between the independent variables on the response value, three-dimensional response surface plots drawn by the Box–Behnken Design were shown in Figure 2a–f. For each figure, the extraction yield of total phenolics was gained by changing two variables while the other two variables remained unchanged [32]. Figure 2a–f shows the effects of the pairwise interaction between the ethanol concentration and extraction temperature on the extraction yield of total phenolics. It can be seen that the extraction yield of total phenolics increased rapidly with increasing ethanol concentration from 60% to about 70%, and followed by a decrease thereafter, meanwhile, the extraction yield of total phenolics increased rapidly when the extraction temperature changed from 50 °C to 62 °C, and decreased afterwards. Similarly, the extraction yield of total phenolics increased greatly with the increase of extraction time from 30 min to about 52 min, whereas it decreased over 52 min. The liquid-to-solid ratio exhibited a weaker effect on the extraction yield of total phenolics, and the extraction yield of total phenolics increased slowly with the increase of liquid-to-solid ratio from 30 mL/g to 36 mL/g, and then declined.

#### 2.2.3. Verification of Predictive Model

The optimal extraction conditions (X_1_ 70.31%, X_2_ 61.94 °C, X_3_ 51.73 min, and X_4_ 35.63 mL/g) were obtained from Figure 2. Under the optimal conditions, a maximum response value (Y) of 96.12 mg GAE/g DM was obtained from the mathematical prediction of the model. Subsequently, to verify the accuracy and practicability of the model equation, verification experiments were performed. The extraction yield of total phenolics from CAB was 95.84 mg GAE/g DM. This fact proved that the model equation was perfectly suitable for the optimization of the UAE process.

### 2.3. Comparison of UAE and HRE

In addition, to evaluate the efficiency of UAE, conventional HRE of total phenolics from CAB was also performed. Compared with UAE, the HRE method had a lower extraction yield of total phenolics (78.32 ± 1.83 mg GAE/g DM). UAE could significantly improve the extraction yield of total phenolics, which could be attributed to the fact that the cavitation effects, thermal effects, and mechanical effects have positive influences on the extraction process [33]. Additionally, compared to previous reports, the extraction yield of total phenolics obtained by UAE in this work was much higher than those obtained by conventional hot water, ethanol, methanol extraction [16].

### 2.4. Analysis of Phenolic Compounds by HPLC

**Figure 3:**
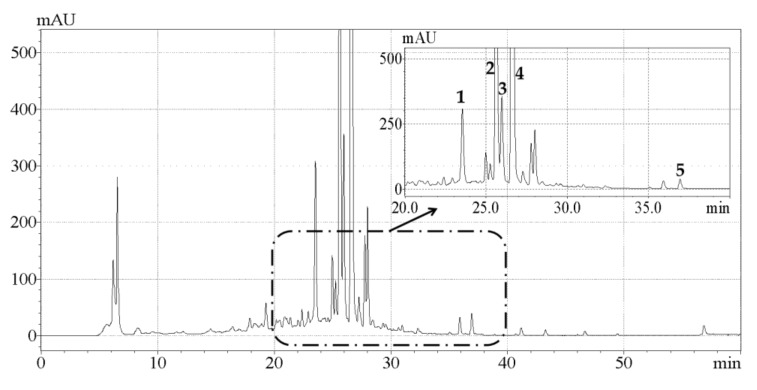
HPLC-DAD chromatogram of the extracts obtained by ultrasonic-assisted extraction under optimized conditions. Peaks 1, 2, 3, 4, and 5 represent eriocitrin, naringin, hesperidin, neohesperidin, and hesperetin, respectively.

A comparative study on the effects of UAE and conventional HRE on the contents of individual phenolic compounds in CAB was conducted. As shown in Figure 3, the five main phenolic compounds detected were hesperetin, hesperidin, naringin, neohesperidin, and eriocitrin, which was in general agreement with previous research [15]. The contents of individual phenolic compounds detected are listed in Table 3, and it was observed that the content of each component extracted by UAE was higher than that obtained by HRE. Naringin contents of 6.17 ± 0.04 and 5.03 ± 0.03 mg/g, hesperidin contents of 10.22 ± 0.06 and 8.25 ± 0.02 mg/g, and neohesperidin contents of 15.45 ± 0.05 and 11.77 ± 0.03 mg/g were quantified respectively, which were very consistent with the result reported by Shen et al. that naringin, hesperidin and neohesperidin are the predominant phenolic compounds in CAB [17]. To our knowledge this was the first study that reported the contents of eriocitrin (1.62 ± 0.03 mg/g) and hesperetin (0.68 ± 0.03 mg/g) in CAB. In addition, Karimi et al. reported the content of quercetin as 0.185 mg/g [16], but we did not actually detect this compound. One possible explanation for this was that the levels of phytochemicals depend on many factors including cultivar variation, environmental condition, and harvest time [34].

### 2.5. Evaluation of Free Radical Scavenging Activities

#### 2.5.1. DPPH· Scavenging Activity

**Figure 4:**
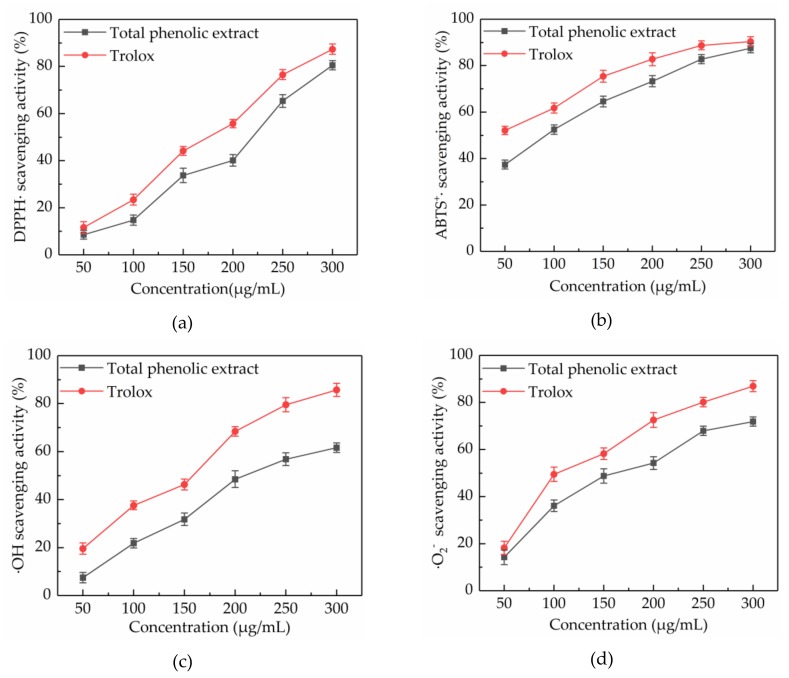
Free radical scavenging activities of total phenolic extract at concentrations of 50, 100, 150, 200, 250, and 300 μg/mL. DPPH· scavenging activity (**a**), ABTS^+^· scavenging activity (**b**), ·OH scavenging activity (**c**) and ·O_2_^−^ scavenging activity (**d**).

Scavenging of DPPH· is a common model used to study the free radical scavenging activity of natural extracts. The DPPH· scavenging activity of a substance is quantitatively related to the number of electrons received by DPPH·. Figure 4a showed that the DPPH· scavenging activity of total phenolic extract from CAB increased in a concentration-dependent manner at concentrations ranging from 50 to 300 μg/mL, throughout slightly lower than trolox. At a concentration of 300 μg/mL, the DPPH· scavenging activity of total phenolic extracts from CAB and trolox were 80.54% ± 1.94% and 91.32% ± 2.21%, respectively. Besides, the IC_50_ values of total phenolic extracts from CAB and trolox were 197.007 and 157.469 μg/mL, respectively. Also, the total phenolic extracts from CAB under the optimal conditions showed a higher DPPH· scavenging activity than methanolic, ethanolic, and aqueous extracts [16].

#### 2.5.2. ABTS^+^· Scavenging Activity

ABTS^+^· scavenging activity assay is another important measure to evaluate the free radical scavenging activity of natural products. ABTS can be oxidized into green ABTS^+^· under the action of oxidants, and the production of ABTS^+^· is inhibited in the presence of antioxidants. ABTS^+^· scavenging activities of total phenolic extract from CAB and trolox are displayed in Figure 4b. It was found that their ABTS^+^· scavenging activities increased with increasing concentration. When the concentration was in the range of 50–300 μg/mL, the ABTS^+^· scavenging activity of total phenolic extract from CAB was slightly weaker than trolox. Additionally, the IC_50_ values were 83.878 and 53.746 μg/mL, respectively, for total phenolic extract from CAB and trolox. Hence, the total phenolic extract from CAB was considered to have a high ABTS^+^· scavenging activity.

#### 2.5.3. ·OH Scavenging Activity

The ·OH radical has very strong oxidation ability due to its extremely effective ability to acquire electrons, which can be generated through the Fenton reaction. Almost all biological molecules are exposed to oxidative damage because of surplus ·OH. Figure 4c shows that the ·OH scavenging activity of total phenolic extract from CAB increased with an increase in concentration. A 300 μg/mL of total phenolic extract from CAB and trolox exhibited 61.65% ± 1.97% and 85.74% ± 2.72% scavenging activity respectively, and their IC_50_ values were 218.643 and 129.665 mg/mL, respectively. Those results indicated that the total phenolic extracts from CAB were good scavengers of ·OH.

#### 2.5.4. ·O_2_^−^ Scavenging Activity

Although ·O_2_^−^ is a weak oxidant, it can cause damage to an organism, because it is ubiquitous and can degrade to other kinds of reactive oxygen species. Hence, scavenging ·O_2_^−^ is also very necessary. As shown in Figure 4d, trolox exhibited a better ·O_2_^−^ scavenging activity than the total phenolic extracts from CAB at concentrations from 50 to 300 μg/mL, and their ·O_2_^−^ scavenging activities were dose-dependent. At a concentration of 300 μg/mL, total phenolic extract from CAB and trolox respectively exhibited 74.84% ± 1.96% and 86.94% ± 2.37%. Besides, the IC_50_ of total phenolic extract from CAB was 158.885 μg/mL, whereas the IC_50_ of trolox was 112.520 μg/mL. These results proved that the total phenolic extract from CAB can scavenge ·O_2_^−^ effectively.

### 2.6. Evaluation of Anti-HMG-CoA Reductase Activity

**Figure 5:**
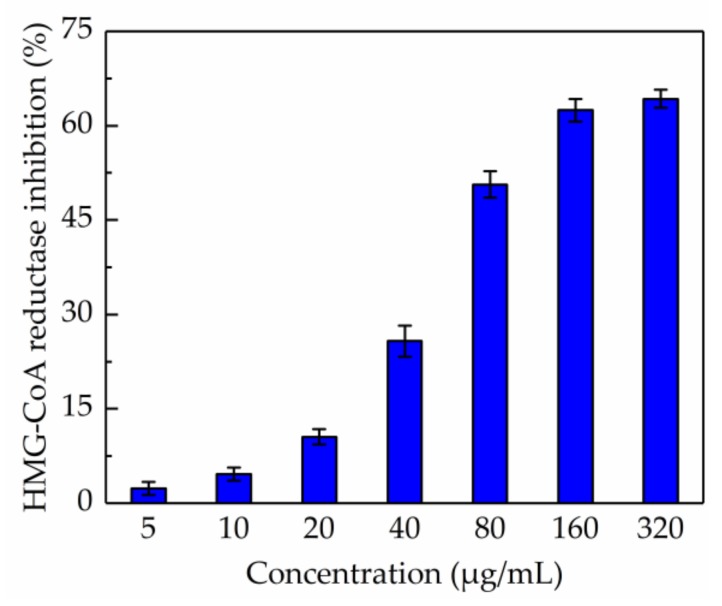
Inhibition of β-hydroxy-β-methylglutaryl-CoA reductase activity by total phenolic extract.

The in vitro test of anti-HMG-CoA reductase activity of the total phenolic extract was carried out and the result is shown in Figure 5. It was demonstrated that the total phenolic extract significantly inhibited HMG-CoA reductase activity when the concentration was higher than 160 µg/mL; the inhibition drastically increased from 2.35% to 64.27% when the concentration of total phenolic extracts was within the range 5–320 µg/mL, and the IC_50_ value was 117.165 µg/mL. Besides, the IC_50_ value measured for pravastatin was 68.54 nM, which was consistent with a previous report [35]. These results suggested that the total phenolic extract from CAB might help in reducing the production of cholesterol.

## 3. Materials and Methods

### 3.1. Chemicals and Reagents

Folin–Ciocalteu, trolox, gallic acid, 1,1-diphenyl-2-picrylhydrazyl (DPPH), 2,2′-azino-bis (3-ethyl-benzothiazoline-6-sulfonic acid) diammonium salt (ABTS), nitroblue tetrazolium (NBT) chloride, *N*-methylphenazonium methyl sulfate (PMS), nicotinamide adenine dinucleotide (NADH) were purchased from Sigma Chemical Co. (St. Louis, MO, USA). Other analytical grades of methanol, ethanol, H_2_O_2_, FeSO_4_, K_2_S_2_O_8_ and salicylic acid were purchased from Tianjin Kemio Chemical Co. (Tianjin, China).

### 3.2. Plant Material

CAB were collected in March 2018, from Jinhua city, Zhejiang province, China. A voucher specimen (DDY-20180301) was deposited at the Key Laboratory of Biotechnology and Bioresources Utilization, Ministry of Education, Dalian, China. The raw material was dried, powdered, and sifted with an 80-mesh sieve, before finally preserving in a desiccator.

### 3.3. UAE of Total Phenolics

#### 3.3.1. Single-Factor Experiments

UAE of total phenolics from CAB was carried out with an ultrasonic apparatus (KQ-5200DE, Kunshan ultrasonic Co. Ltd., Suzhou, China), and ultrasound frequency and ultrasound power were set as 40 kHz and 100 W, respectively [36,37]. Each 5 g of sample powder was placed into a volumetric flask (250 mL), then extracted with various ethanol concentrations (30%, 40% 50%, 60%, 70%, 80%), temperatures (30, 40, 50, 60, 70, 80 °C), liquid-solid ratio (15, 20, 25, 30, 35, 40 mL/g) for various times (15, 30, 45, 60, 75, 90 min) respectively. The extracts were filtered, combined, after which the extraction solvent was replenished to bring the final volume of the extract to 250 mL. The extraction yield of total phenolics from CAB for each extraction experiment was expressed as mg of gallic acid equivalent (GAE) on g of dry matter (DM).

#### 3.3.2. Box–Behnken Design

The Box–Behnken is an efficient design for response surface methodology, which has been widely applied to optimize the extraction process of natural active substances. In this work, based on single-factor experiments, a Box–Behnken design with four factors (ethanol concentration X_1_, extraction temperature X_2_, extraction time X_3,_ and liquid-solid ratio X4) and three levels (−1, 0, +1) including 29 experimental runs, was used to evaluate the combined effects on the extraction yield of total phenolics. A second-order polynomial model was used to describe the mathematical relationship between independent variables and response values.

(2) $$Y=\alpha_{0}+\sum_{i=1}^{4}\alpha_{i}X_{i}+\sum_{i=1}^{4}\alpha_{ii}X_{i}{}^{2}+\sum_{i=1}^{3}\sum_{j=i+1}^{4}\alpha_{ij}X_{i}X_{j}$$

where *Y* is the response variable, *X*_i_ and *X*_j_ are independent variables, α_0_ is a constant coefficient, *α*_1_, *α*_2,_ and *α*_3_ represent the linear coefficient, interaction coefficient of two variables and quadratic coefficient of one variable, respectively.

### 3.4. HRE of Total Phenolics from CAB

HRE of total phenolics from CAB was performed under the optimal UAE conditions with slightly modification. Briefly, 5 g of sample powder was kept in a thermostatic water bath at 62 °C and extracted three times with 175 mL of 70% ethanol under reflux, each time for 52 min. After HRE, the extracts were processed by the method described in Section 3.3.1.

### 3.5. Determination of Total Phenolic Content

The measurement of total phenolics content was conducted using the Folin–Ciocalteu colorimetric method as described by Seifzadeh et al. [38]. Briefly, 0.25 mL of total phenolic extract solution was blended with 1.25 mL of the Folin–Ciocalteu reagent (diluted 10-fold by deionized water), then the mixture was neutralized by the addition of 1 mL of 7.5% Na_2_CO_3_. After one hour of incubation at 45 °C in the dark, the absorbance was recorded at 765 nm. Gallic acid was used as a standard. The total phenolics content in the extracts was calculated using the linear regression equation:

(3) $$A=0.1265\;C+0.0228\;(n\;=\;6,\;R^{2}\;=\;0.9997)$$

where *A* was the absorbance of the sample, *C* the final total phenolics concentration.

### 3.6. HPLC Analysis

The extracts obtained from UAE under optimized conditions and conventional HRE were analyzed by an HPLC system (Shimadzu, Kyoto, Japan), which was equipped with two LC-20AD pumps and a diode array detector (SPD-M20A). All chromatography experiments were performed on a YMC-Pack ODS-A C18 (250 mm × 4.6 mm, 5 μm). The mobile phase was comprised of solvent A (0.1% TFA in water) and solvent B (acetonitrile). The solvent gradient in volume ratios was as follows: 0–60 min, 10–100% B. The system was operated at a flow rate of 0.5 mL/min. Analyses were carried out at room temperature, the detection wavelength was set at 280 nm. The phenolic compounds including hesperetin, hesperidin, naringin, neohesperidin, and eriocitrin were identified by comparison with the retention time and UV spectra of individual standards, and quantified using the external standard method.

### 3.7. Free Radical Scavenging Activity Assay

#### 3.7.1. DPPH· Scavenging Activity

The DPPH· scavenging activity assay was operated as the procedure described by Shen et al. [39] with slight modifications. Briefly, 3.2 mL of freshly prepared DPPH solution (0.2 mM in ethanol) and 0.4 mL of phenolic extracts in ethanol at different concentrations (50–300 μg/mL) were mixed and shaken well in the dark at room temperature. After 60 min, the absorbance was measured at 517 nm using a spectrophotometer (UV-2600, Shimadzu, Japan). The DPPH· scavenging activity of trolox was also analyzed for positive control. The percentage of DPPH· scavenging was calculated by the following equation:

(4) $$\mathrm{DPPH}\cdot \;\mathrm{scavenging}\;\mathrm{activity}\;(\%)=(1-\frac{A_{\mathrm{sample}}}{A_{\mathrm{blank}}})\times 100$$

where *A*_sample_ was the absorbance of the total phenolic extract, and the *A*_balnk_ was the absorbance of the sample without phenolic extract.

#### 3.7.2. ABTS^+^· Scavenging Activity Assay

The ABTS^+^· scavenging activity assay was conducted as in the method described by a previous study with slight modifications [40]. Briefly, a redox reaction was triggered by blending the ABTS solution (7.0 mM) and K_2_S_2_O_8_ solution (2.45 mM) in a ratio of 1:1. After 16 h, the reaction solution was adjusted with ethanol to yield an absorbance between 0.68 and 0.72 at 734 nm. Then, 125 μL of each sample (50, 100, 150, 200, 250 and 300 μg/mL) was mixed with 200 μL of ABTS^+^· solution. After one minute, the absorbance was recorded at 734 nm. Trolox was used as a standard. The percentage of ABTS^+^· scavenging was calculated using the following equation:

(5) $${\mathrm{ABTS}}^{+}\cdot \;\mathrm{scavenging}\;\mathrm{activity}\;(\%)=(1-\frac{A_{\mathrm{sample}}}{A_{\mathrm{blank}}})\times 100$$

where *A*_sample_ was the absorbance of the total phenolic extract, and the *A*_balnk_ was the absorbance of a blank (without total phenolic extract).

#### 3.7.3. ·OH Scavenging Activity Assay

The ·OH scavenging activity of total phenolic extract was measured according to the method reported previously with some modifications [41]. The ·OH was obtained through a Fenton reaction. Explicitly, the reaction system consisted of 1.0 mL FeSO_4_ (9.0 mM), 1.0 mL H_2_O_2_ (9.0 mM), and 1.0 mL of total phenolic extract at different concentrations (50, 100, 150, 200, 250, and 300 mg/mL), then 1.0 mL of salicylic acid (3.0 mM) was added to initiate the reaction. The reaction mixture was incubated at 37 °C for one hour, and the absorbance was measured at 510 nm. Trolox was used as a positive control. The ·OH scavenging activity was measured using the following equation:

(6) $$\cdot \mathrm{OH}\;\mathrm{scavenging}\;\mathrm{activity}\;(\%)=(1-\frac{A_{1}-A_{2}}{A_{0}})\times 100$$

where, *A*_0_, *A*_1_, and *A*_2_ are the absorbance of the blank control (without total phenolic extract), the absorbance of total phenolic extract and the absorbance without H_2_O_2_, respectively.

#### 3.7.4. ·O_2_^−^ Scavenging Activity Assay

The ·O_2_^−^ scavenging activity of total phenolic extract was measured based on the modified method described previously [42]. Briefly, the reaction system consisted of 3 mL of Tris-HCl buffer solution (16 mM, pH 8.0) containing 1 mL of NBT (50 μM), 1 mL of NADH (78 μM), and 1 mL of total phenolic extract at different concentrations (50, 100, 150, 200, 250 and 300 μg/mL). Then 1.0 mL of PMS (10 μM) was added into the system to initiate the reaction. The mixture was maintained at 25 °C for five minutes, followed by measurement of the absorbance at 560 nm. Trolox was used as a comparison. The ·O_2_^−^ scavenging activity was measured using the following equation:

(7) $$\cdot O_{2}{}^{-}\;\mathrm{scavenging}\;\mathrm{activity}\;(\%)=(1-\frac{A_{b}}{A_{s}})\times 100$$

where *A*_s_ and *A*_b_ are the absorbance of the total phenolic extract and the absorbance of the blank control (without total phenolic extract), respectively.

### 3.8. Anti-HMG-CoA Reductase Activity Assay

HMG-CoA reductase inhibitory activity of the total phenolic extract from CAB was evaluated with the commercially available HMG-CoA reductase assay kit purchased from Sigma-Aldrich (St. Louis, MO, USA) [43,44]. The specific operation method was in accordance with the instruction: each well of 96-well plate containing 1 µL of different concentrations of total phenolic extract, 4 µL of NADPH (final concentration of 400 μM), 12 μL of HMG-CoA substrate (final concentration of 300 μg/mL), followed by the addition of phosphate buffer (pH 7.4) to achieve a final volume of 200 µL. Then, 2 μL of the HMG-CoA reductase was added into each well to activate the reaction system. Immediately, the 96-well plate was shaken mechanically in a microplate reader (BioTek, Synergy H1, Winooski, VT, USA) for 10 s. The consumption rate of NADPH was measured once at 20 s for up to 600 s by recording the absorbance of the reaction system at 340 nm. Pravastatin was used as positive control. The HMG-CoA reductase inhibition (%) was calculated using the following formula:

(8) $$\mathrm{HMG}-\mathrm{CoA}\;\mathrm{reductase}\;\mathrm{inhibition}\;(\%)=\frac{\Delta A_{\mathrm{control}}-\Delta A_{\mathrm{test}}}{\Delta A_{\mathrm{control}}}\times 100$$

where *A*_test_ and *A*_control_ are the absorbance of the total phenolic extract and the absorbance of control (without total phenolic extract), respectively

### 3.9. Statistical Methods

The data were reported as the mean ± standard deviation (SD) from three parallel experiments. The half maximal inhibitory concentration (IC_50_) was calculated using the linear regression method by the SPSS 24.0 software (SPSS Inc., Chicago, IL, USA). Design-Expert 10 (Stat-Ease Inc., Minneapolis, MN, USA) was used for designing the experiments and statistical analysis.

## 4. Conclusions

In the present work, the optimum UAE technology to obtain total phenolics from CAB was successfully developed through single-factor experiments coupled with the Box–Behnken design. The optimum parameters including ethanol concentration, ultrasonic temperature, ultrasonic time, liquid-to-solid ratio were 70.31%, 61.94 °C, 51.73 min and 35.63 mL/g, respectively. Under these conditions, the extraction yield of total phenolics from CAB was 95.84 mg GAE/g DM, which was close to the theoretical value (96.12 mg GAE/g DM). Based on the HPLC analysis, UAE under optimized conditions was a more efficient method for extracting phenolic compounds including eriocitrinnaringin, hesperidin, neohesperidin, and hesperetin compared with conventional HRE. In addition, the study on free radical scavenging activities indicated that the total phenolic extract obtained under the optimum condition had excellent scavenging effects on DPPH·, ABTS^+^·, ·OH, and ·O_2_^−^, with the corresponding IC_50_ values of 197.007, 83.878, 218.643, and 158.885 μg/mL, while the total phenolic extract also exhibited good potential to inhibit HMG-CoA reductase in vitro, with an IC_50_ value of 117.165 μg/mL. Therefore, the study is conducive for the optimum utilization of CAB as a great source of total phenolics with good free radical scavenging, anti-HMG-CoA reductase activities.

## Figures and Tables

**Table 1:** Box–Behnken design matrix and response values for the extraction yield of total phenolics.

Run	Independent Variables				Y (mg GAE/g DM)	
	X_1_ (%)	X_2_ (°C)	X_3_ (min)	X_4_ (mL/g)	Experimental	Predicted
1	60	60	45	30	72.19	73.63
2	70	60	45	35	95.81	94.49
3	70	60	45	35	95.37	94.49
4	70	70	45	30	79.37	78.38
5	70	50	60	35	74.97	76.29
6	80	60	30	35	72.36	73.48
7	70	60	30	30	77.98	76.20
8	60	60	30	35	68.28	70.01
9	80	60	45	30	77.99	79.74
10	70	60	30	40	82.47	82.40
11	70	50	45	40	72.52	73.68
12	60	50	45	35	65.69	64.30
13	70	60	45	35	93.64	94.49
14	70	60	45	35	93.47	94.49
15	70	70	45	40	85.27	85.21
16	70	60	60	30	90.21	89.55
17	60	60	60	35	81.90	80.96
18	80	70	45	35	74.01	74.67
19	70	60	45	35	94.17	94.49
20	80	50	45	35	69.86	68.77
21	80	60	60	35	84.4	82.84
22	60	60	45	40	81.27	80.07
23	70	60	60	40	88.32	89.37
24	70	70	60	35	86.22	87.01
25	70	50	30	35	69.39	69.15
26	80	60	45	40	80.19	79.31
27	70	50	45	30	74.26	74.50
28	70	70	30	35	74.61	73.84
29	60	70	45	35	73.43	73.79

**Table 2:** ANOVA for Box–Behnken design.

Source	Sum of Squares	df	Mean Square	*F*-Value	*p*-Value
Model	2196.5259	14	156.8947	66.3436	<0.0001 ***
X_1_	21.4669	1	21.4669	9.0774	0.0093 **
X_2_	178.0240	1	178.0240	75.2782	<0.0001 ***
X_3_	309.3721	1	309.3721	130.8193	<0.0001 ***
X_4_	27.1201	1	27.1201	11.4679	0.0044 **
X_1_X_2_	3.2220	1	3.2220	1.3624	0.2626
X_1_X_3_	0.6241	1	0.6241	0.2639	0.6154
X_1_X_4_	11.8336	1	11.8336	5.0039	0.0421 *
X_2_X_3_	9.0902	1	9.0902	3.8438	0.0701
X_2_X_4_	14.5924	1	14.5924	6.1705	0.0263 *
X_3_X_4_	10.1761	1	10.1761	4.3030	0.0570
X_1_^2^	923.4078	1	923.4078	390.4671	<0.0001 ***
X_2_^2^	961.9172	1	961.9172	406.7509	<0.0001 ***
X_3_^2^	213.6335	1	213.6335	90.3359	<0.0001 ***
X_4_^2^	124.0230	1	124.0230	52.4437	<0.0001 ***
Residual	33.1083	14	2.3649		
Lack of Fit	28.7262	10	2.8726	2.6222	0.1829
Pure Error	4.3821	4	1.0955		
Cor Total	2229.6342	28			

*R*^2^ = 0.9852, Adj. *R*^2^ = 0.9703, C.V% = 1.91, * *p* < 0.05, ** *p* < 0.01, *** *p* < 0.001.

**Table 3:** Comparison of individual phenolic compounds contents in CAB extracted by UAE and HRE.

Extraction Method	Content of Individual Phenolic Compounds (mg/g)				
	Eriocitrin	Naringin	Hesperidin	Neohesperidin	Hesperetin
UAE	1.62 ± 0.03	6.17 ± 0.04	10.22 ± 0.03	15.45 ± 0.05	0.68 ± 0.03
HRE	1.17 ± 0.02	5.03 ± 0.03	8.25 ± 0.02	11.77 ± 0.03	0.56 ± 0.01
